# Supplementary material for: Drug repurposing for aging research using model organisms
Source: Aging Cell. 2017 Jun 16;16(5):1006–15. doi: 10.1111/acel.12626 (PMC5595691; doi:10.1111/acel.12626)
Supplement: Supplementary file 7 — Data S1 Zip‐Archive of all report cards. [file ACEL-16-1006-s007.zip › RC_8PR.pdf]

8PR

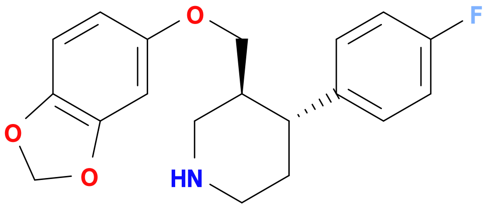

Database identifiers

ChEMBLCompound	CHEMBL490
DrugBank	DB00715
CHEBI	7936
eMolecules	1935652

Ranking

	Rank	Score
Drosophila	209/697	0.694
C. elegans	75/591	0.358

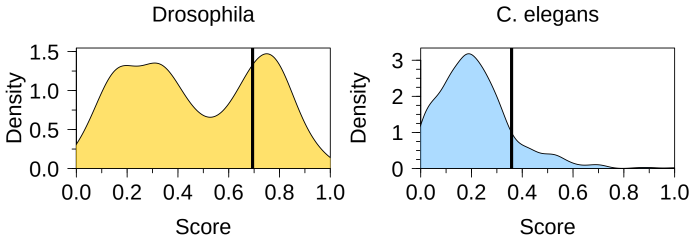

	Ageing implication	Domain conservation	Binding site conservation	Binding affinity	Bioavailability	Lipinski	Promiscuity	Purchasability	Drug approval	**Total**
Drosophila	0.624	0.969	1.0	0.908	(0.9)	0.0	-0.0	0.1	0.1	0.694
C. elegans	0.624	0.968	1.0	0.908	0.289	0.0	-0.0	0.1	0.1	0.358

Names

- **paroxetine**
- (-)-(3S,4R)-4-(p-fluorophenyl)-3-((3,4-(methylenedioxy)phenoxy)methyl)piperidine
- (3S-trans)-3-((1,3-benzodioxol-5-yloxy)methyl)-4-(4-fluorophenyl)piperidine
- Paxil
- Pexeva

Roles

anxiolytic drug, serotonin uptake inhibitor, hepatotoxic agent, P450 inhibitor, antidepressant

Status

Approved drug (according to ChEMBL)	Yes
Administration Route	Oral
Number of Rule of 5 violations	0
Binding affinity to original target in log units (RF-Score prediction)	7.3
Burns *C. elegans* bioavailability prediction	-0.69

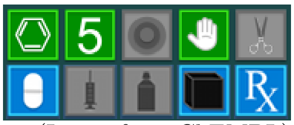

(Image from ChEMBL)

Medical Information

Indicatation: Labeled indications include: major depressive disorder (MDD), panic disorder with or without agoraphobia, obsessive-compulsive disorder (OCD), social anxiety disorder (social phobia), generalized anxiety disorder (GAD), post-traumatic stress disorder (PTSD), and premenstrual dysphoric disorder (PMDD). Unlabeled indications include: eating disorders, impulse control disorders, vasomotor symptoms of menopause, obsessive-compulsive disorder (OCD) in children, and mild dementia-associated agitation in nonpsychotic individuals. Brisdelle, which consists of paroxetine mesylate is indicated for the treatment of moderate to severe vasomotor symptoms (like hot flashes) associated with menopause.
Mechanism of action: Paroxetine is a potent and highly selective inhibitor of neuronal serotonin reuptake. Paroxetine likely inhibits the reuptake of serotonin at the neuronal membrane, enhances serotonergic neurotransmission by reducing turnover of the neurotransmitter, therefore it prolongs its activity at synaptic receptor sites and potentiates 5-HT in the CNS; paroxetine is more potent than both sertraline and fluoxetine in its ability to inhibit 5-HT reuptake. Compared to the tricyclic antidepressants, SSRIs have dramatically decreased binding to histamine, acetylcholine, and norepinephrine receptors. The mechanism of action for the treatment of vasomotor symptoms is unknown.
Toxicity: LD50=500mg/kg (orally in mice). Symptoms of overdose include: coma, dizziness, drowsiness, facial flushing, nausea, sweating, tremor, vomiting. Side effects include: nervous system effects such as asthenia, somnolence, dizziness, insomnia, tremor, and nervousness; GI effects such as nausea, decreased appetite, constipation, diarrhea, and dry mouth; impotence, ejaculatory dysfunction (principally ejaculatory delay), and other male genital disorders; female genital disorders (principally anorgasmia or difficulty reaching climax/orgasm); and sweating. Discontinuation syndrome may occur with abrupt withdrawal. Symptoms of discontinuation syndrome include flu-like symptoms, insomnia, nausea, imbalance, sensory changes, and hyperactivity.
Metabolism: Paroxetine is extensively metabolized after oral administration, likely in the liver. The main metabolites are polar and conjugated products of oxidation and methylation, which are readily eliminated by the body. The predominant metabolites are glucuronic acid and sulfate conjugates. Paroxetine metabolites do not possess significant pharmacologic activity (less than 2% that of parent compound). Paroxetine is metabolized by cytochrome P450 (CYP) 2D6. Enzyme saturation appears to account for the nonlinear pharmacokinetics observed with increasing dose and duration of therapy.
Retail: Advanced Pharmaceutical Services Inc., Aidarex Pharmacuticals LLC, Alphapharm Party Ltd., Amerisource Health Services Corp., Apotex Inc., A-S Medication Solutions LLC and 53 more suppliers
Apo-Paroxetine 20 mg Tablet - 1.05 USD / tablet
Co Paroxetine 20 mg Tablet - 1.05 USD / tablet
Mylan-Paroxetine 20 mg Tablet - 1.05 USD / tablet
Novo-Paroxetine 20 mg Tablet - 1.05 USD / tablet
Phl-Paroxetine 20 mg Tablet - 1.05 USD / tablet
Pms-Paroxetine 20 mg Tablet - 1.05 USD / tablet
and 39 more options

(Information from DrugBank)

Compound Target Characteristics

Beta-adrenergic receptor kinase 1

Best gene implication in ageing for this target family came from gene F1LMA4 via mapping the annotation from RGD 2062 annotated in RGD 2014-03-11. Annotation GO 7568 (aging) was Inferred from Expression Pattern

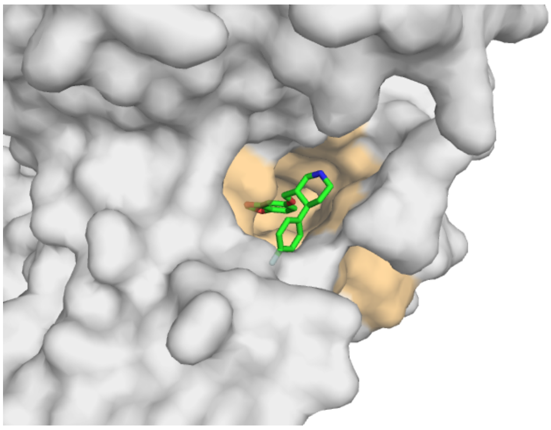

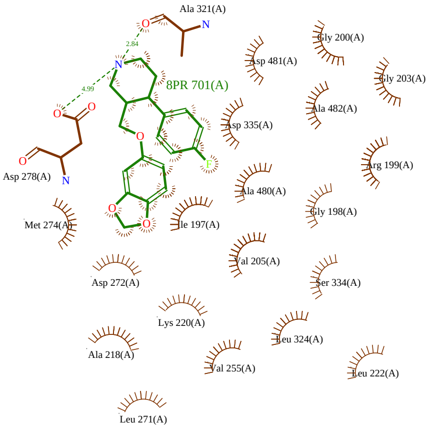

protein	amino acids contacts (binding site)
PDB:3v5w:chainA:P25098	I G R G G V A K L V L D M D A L S D A D A
sp:P25098:ARBK1_HUMAN	I G R G G V A K L V L D M D A L S D A D A
tr:F1LMA4:F1LMA4_RAT	I G R G G V A K L V L D M D A L S D A D A
tr:Q7TS64:Q7TS64_MOUSE	I G R G G V A K L V L D M D A L S D A D A
tr:Q3U1V3:Q3U1V3_MOUSE	I G R G G V A K L V L D M D A L S D A D A
sp:Q99MK8:ARBK1_MOUSE	I G R G G V A K L V L D M D A L S D A D A
tr:E1JGX2:E1JGX2_DROME	I G R G G V A K L V L D M D A L S D A D A
sp:P32865:GPRK1_DROME	I G R G G V A K L V L D M D A L S D A D A
sp:Q09639:GRK2_CAEEL	I G R G G V A K L V L D M D A L S D A D A

	whole protein		domain-based		contact-based	
protein	ident	simil	ident	simil	ident	simil
PDB:3v5w:chainA:P25098	1.0	1.0	1.0	1.0	1.0	1.0
sp:P25098:ARBK1_HUMAN	1.0	1.0	1.0	1.0	1.0	1.0
tr:F1LMA4:F1LMA4_RAT	0.94	0.94	1.0	1.0	1.0	1.0
tr:Q7TS64:Q7TS64_MOUSE	0.93	0.94	1.0	1.0	1.0	1.0
tr:Q3U1V3:Q3U1V3_MOUSE	0.99	1.0	1.0	1.0	1.0	1.0
sp:Q99MK8:ARBK1_MOUSE	0.99	1.0	1.0	1.0	1.0	1.0
tr:E1JGX2:E1JGX2_DROME	0.64	0.87	0.83	0.94	1.0	1.0
sp:P32865:GPRK1_DROME	0.64	0.87	0.83	0.94	1.0	1.0
sp:Q09639:GRK2_CAEEL	0.62	0.85	0.8	0.94	1.0	1.0

Gprk1 (FBgn0260798) associated phenotypes

heat sensitive, neurophysiology defective

(Information from FlyBase)

Gprk1 (UniProt:P32865) annotation

Function: Specifically phosphorylates the activated forms of G protein-coupled receptors.

(Information from UniProt)

grk-2 (WBGene00001709) associated phenotypes

aqueous negative chemotaxis variant, bitter tastant response variant, copper response variant, diacetyl chemotaxis defective, isoamyl alcohol chemotaxis defective, octanol chemotaxis defective, osmotic avoidance defective, pyrazine chemotaxis defective, quinine chemotaxis defective

(Information from WormBase)

grk-2 (UniProt:Q09639) annotation

Function: Specifically phosphorylates the activated forms of G protein-coupled receptors.

(Information from UniProt)
